# Genetic resources of common ash (*Fraxinus excelsior* L.) in Poland

**DOI:** 10.1186/s12870-024-04886-z

**Published:** 2024-03-13

**Authors:** Joanna Meger, Czesław Kozioł, Małgorzata Pałucka, Jarosław Burczyk, Igor J. Chybicki

**Affiliations:** 1 Department of Genetics, Faculty of Biological Sciences, Kazimierz Wielki University, Chodkiewicza 30, Bydgoszcz, 85-064 Poland; 2 Szklarska Poręba Forest District, Krasińskiego 6, Szklarska Poręba, 58-580 Poland; 3 Kostrzyca Forest Gene Bank, Miłków 300, Miłków, 58-535 Poland

**Keywords:** Common ash, Conservation, Genetic resources, Microsatellite markers, Forest management

## Abstract

**Background:**

Knowledge of genetic structure and the factors that shape it has an impact on forest management practices. European ash (*Fraxinus excelsior* L.) has declined dramatically throughout its range as a result of a disease caused by the fungus *Hymenoscyphus fraxineus*. Despite the need for conservation and restoration of the species, genetic data required to guide these efforts at the country level are scarce. Thereofore, we studied the chloroplast and nuclear genetic diversity of 26 natural common ash populations (1269 trees) in Poland.

**Results:**

Chloroplast polymorphisms grouped the populations into two geographically structured phylogenetic lineages ascribed to different glacial refugia (the Balkans and the Eastern Alps). However, the populations demonstrated high genetic diversity (mean *A*_*R*_ = 12.35; mean *H*_*o*_ = 0.769; mean *H*_*e*_ = 0.542) but low differentiation based on nuclear microsatellites (*F*_*ST*_ = 0.045). Significant spatial genetic structure, consistent with models of isolation by distance, was detected in 14 out of 23 populations. Estimated effective population size was moderate-to-high, with a harmonic mean of 57.5 individuals per population.

**Conclusions:**

Genetic diversity was not homogeneously distributed among populations within phylogenetic gene pools, indicating that ash populations are not equal as potential sources of reproductive material. Genetic differences among populations could be related to their histories, including founder effects or gene flow between evolutionary lineages (admixture). Our results suggest that ash stands across Poland could be treated as two main management units (seed zones). Therefore, despite the homogenizing effect of pollen gene flow known for this species, the genetic structure should be taken into account in the management of the genetic resources of the common ash. Although ash dieback poses an additional challenge for the management of genetic resources, efforts should be directed towards protecting populations with high genetic diversity within defined phylogenetic units, as they may be an important source of adaptive variation for future stands.

**Supplementary Information:**

The online version contains supplementary material available at 10.1186/s12870-024-04886-z.

## Background

Forests provide enormously important ecosystem goods and services and play a fundamental role in biodiversity conservation [1]. Forests cover nearly 30% of the Earth’s land area [2], containing 80% of terrestrial biodiversity [3]. Today, however, forests face a growing array of threats related to climate change, air pollution, pests and diseases, urbanization, and forest fragmentation. These threats are expected to shape species composition, and may adversely affect genetic diversity and reduce the future adaptive potential of forest trees, and forest ecosystems in general [4]. Therefore, to maintain the sustainability of forest resources, active conservation and protection are currently the key forest management objectives.

There are two main approaches to maintaining forest biodiversity. On the one hand, it is important to ensure the maintenance of populations within their native environments, to which they are presumably adapted. On the other hand, it is crucial to conserve genetic variation outside native habitats, establishing progeny plantations, and gene or seed banks [5]. However, because so much habitat has been and continues to be lost, these conservation methods may be insufficient to ensure the survival of a large number of species and populations. To this end, whole-habitat restoration, species reintroduction, and population augmentation are becoming increasingly important conservation tools. Given that plant populations are often adapted to local site conditions, identifying geographic guidelines for seed transfer is a crucial consideration in any restoration effort [6].

Many methods have been proposed to define seed transfer zones for different species and at different spatial scales, resulting in several delineation strategies. The ecoregional approach integrates geographic distance [7] and topographic, climatic, or edaphic data to define zones of ecological similarity, within which seeds may be transferred [8, 9]. However, seed zone delineation based on simple ecological clues [10, 11] neglects the fact that environmental similarity does not necessarily equate to genetic similarity or shared evolutionary history. Therefore, optimal seed-zone systems incorporate the available genetic information, which allows for designing reasonable seed transfer guidelines [12].

Neutral molecular markers are often used to define seed transfer zones. Although they do not reflect the action of natural selection and adaptation [13], they nevertheless allow us to determine the overall genetic diversity and the extent of gene flow among populations [14]. As a result, it is possible to identify the natural boundaries of demographic units [15] and define their characteristics, such as effective population size, allelic richness, inbreeding and demographic history. These parameters may help assess the relative importance of individual seed sources in activities for the conservation of a species’ genetic resources.

The common ash (*Fraxinus excelsior* L.) is a temperate tree species with a wide distribution throughout Europe. It occurs as an admixture tree species in various forest communities but rarely becomes a dominant tree [16]. Ash is a valuable broadleaved tree due to its ecological characteristics, excellent wood properties, and high economic value. In Europe, ash has survived the glacial periods in refugia located in the Iberian and Italian Peninsulas, the eastern Alps, and the Balkan Peninsula [17]. As revealed based on nuclear microsatellite markers, postglacial recolonization and extensive pollen-mediated gene flow led to a large Central European gene pool, extending from the British Isles to Lithuania, while distinct gene pools and higher population differentiation were found in southeastern Europe [18]. Interestingly, allelic richness and genetic diversity were low in populations from southeastern Europe, but high in western and central Europe [18]. However, little is known about the genetic diversity of ash populations in Poland, despite their central location within the species range.

Recently, ash populations in Europe have been in decline due to the ash dieback caused by an invasive and swiftly spreading fungus *Hymenoscyphus fraxineus* (T. Kowalski) Baral, Queloz, Hosoya comb. nov [19]. . This pathogen causes severe ash tree mortality, especially in central and eastern Europe [20–22], including Poland, where the pathogen’s negative impact was first identified in 1992 [23]. . Scientists agree that ecosystems suitable for the common ash require immediate restoration to reduce the effects of ash dieback [24]. Even if natural regeneration may suffice to maintain ash populations [25], stands with compromised seed productivity or those that need to be regenerated quickly require artificial regeneration methods based on a properly selected genetic material [26].

Conserving and restoring threatened plant species entails the collection of vast quantities of seeds. To conserve in-situ forest genetic resources of the common ash in Poland following the emergence of ash dieback, 12 natural populations with a high level of genetic diversity were originally included in EUFGIS, i.e., the European system for forest genetic resources. At the same time, seeds and vegetative tissues originating from 122 Polish common ash populations have been stored *ex-situ* in the Kostrzyca Forest Gene Bank, both in -10˚C as well as vapors of liquid nitrogen. However, the current efforts to protect common ash in Poland are hampered by insufficient knowledge of the state and trends of genetic diversity. Therefore, adhering to information on the genetics of common ash in Poland is needed to define seed transfer guidelines for the species’ conservation and restoration. Because the common ash spans several countries, this information might be more widely useful if seed transfer is going to be performed among neighboring countries. The general objective of our study was to characterize the genetic diversity and population genetic structure of common ash in Poland. Our specific objectives were to: [1] investigate the patterns of maternal lineages among the study populations, [2] assess the degree of inbreeding and the effective population size, and [3] investigate the demographic history of common ash in Poland. Since the continuous decline of ash populations in Europe and the potential loss of genetic diversity due to ash dieback, the identification of genetically diverse populations of common ash will be important for future conservation management and the establishment of breeding programs regarding pathogen resistance in this species.

## Results

### Chloroplast microsatellites

#### Genetic variation at chloroplast microsatellites

Three (*ccmp5*, *ccmp6*, *ccmp10*) of the five chloroplast microsatellite loci were polymorphic (Table S1), with the observed size variants yielding six different haplotypes (Table S1). The within-population variation at cp.SSRs was generally low, with the average number (± SD) of 2.08 (± 0.18) haplotypes, the effective number of 1.40 (± 0.09) haplotypes per population, and the average genetic diversity of 0.225 (± 0.042). Details of chloroplast haplotype variation in populations are given in Table S2.

#### Phylogenetic relationships among haplotypes

**Fig. 1:**
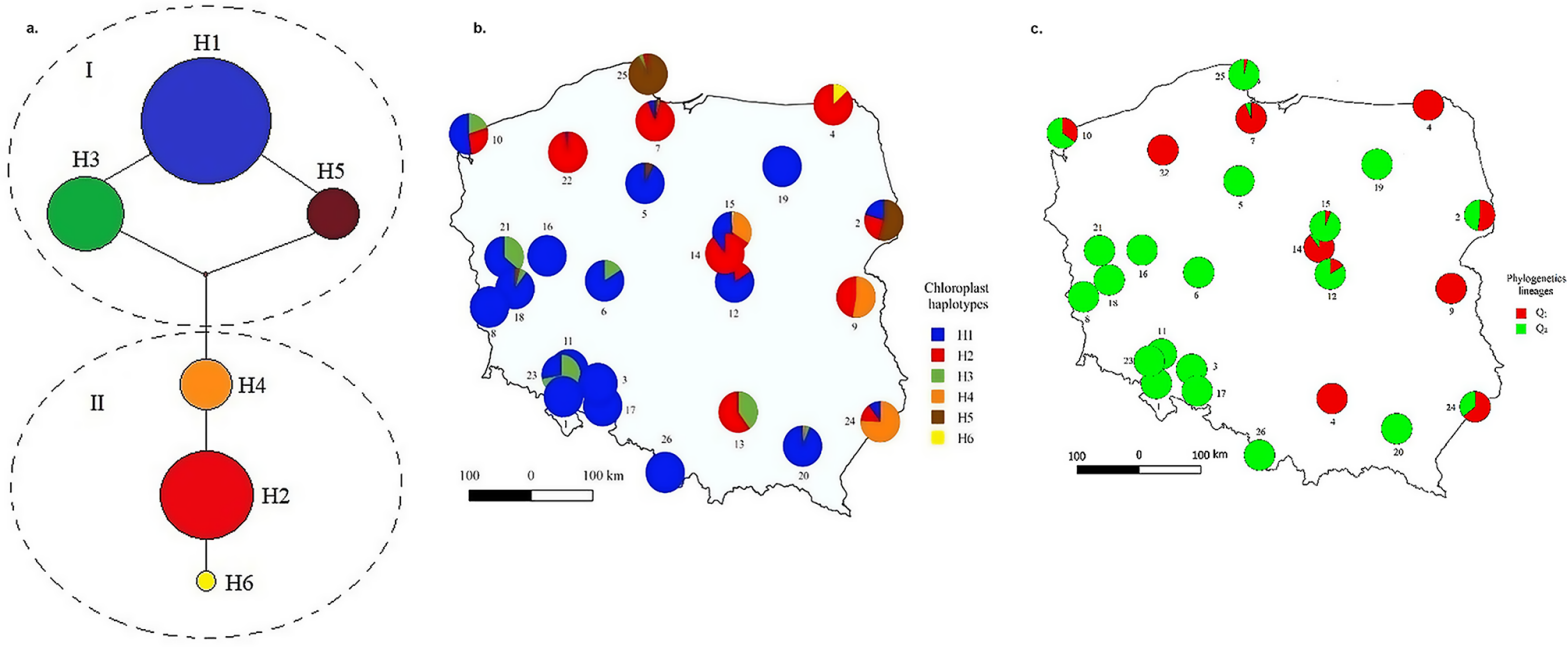
Statistical parsimony network among haplotypes (a). Geographic distribution of chloroplast haplotypes in 26 populations of common ash (b). Phylogeographic structure of the common ash in Poland defined byBAPS software (c)

The haplotype network presented in Fig. 1a indicated the presence of two main groups: the first clade with the three haplotypes (*H1*, *H3*, *H5*) and the cumulative frequency of 72.2%, and the second clade (*H2*, *H4*, *H6*) with the total haplotype frequency of 27.8%.

#### Phylogenetic relationship between populations

Genetic differentiation statistics (*G*_*ST*_ = 0.696 ± 0.020 and *R*_*ST*_ = 0.729 ± 0.008) indicated strong population structuring in ash populations in Poland. The analysis of molecular variance (AMOVA) revealed that 69.5% of the total genetic variation is attributed to genetic differences among ash populations, and 30.5% was ascribed to genetic differentiation between individuals within populations. The neighbor-joining tree also confirmed the presence of differentiation between populations as well as a certain degree of structuring into two groups (Figure S1). Populations in the northeast of Poland (Gołdap, Kolbudy, Międzyrzec, Pińczów, Płock, Szczecinek, and Tomaszów) formed the first group, while a majority of the southwestern populations formed the second group (Bardo Śląskie, Browsk, Brzeg, Jamy, Jarocin, Lubsko, Międzyzdroje, Miękinia, Niepołomice, Płońsk, Pniewy, Prudnik, Przytok, Spychów, Strzyżów, Sulęcin, Świdnica, Wejherowo and Wisła).

#### Identification of phylogenetic lineages

**Fig. 2:**
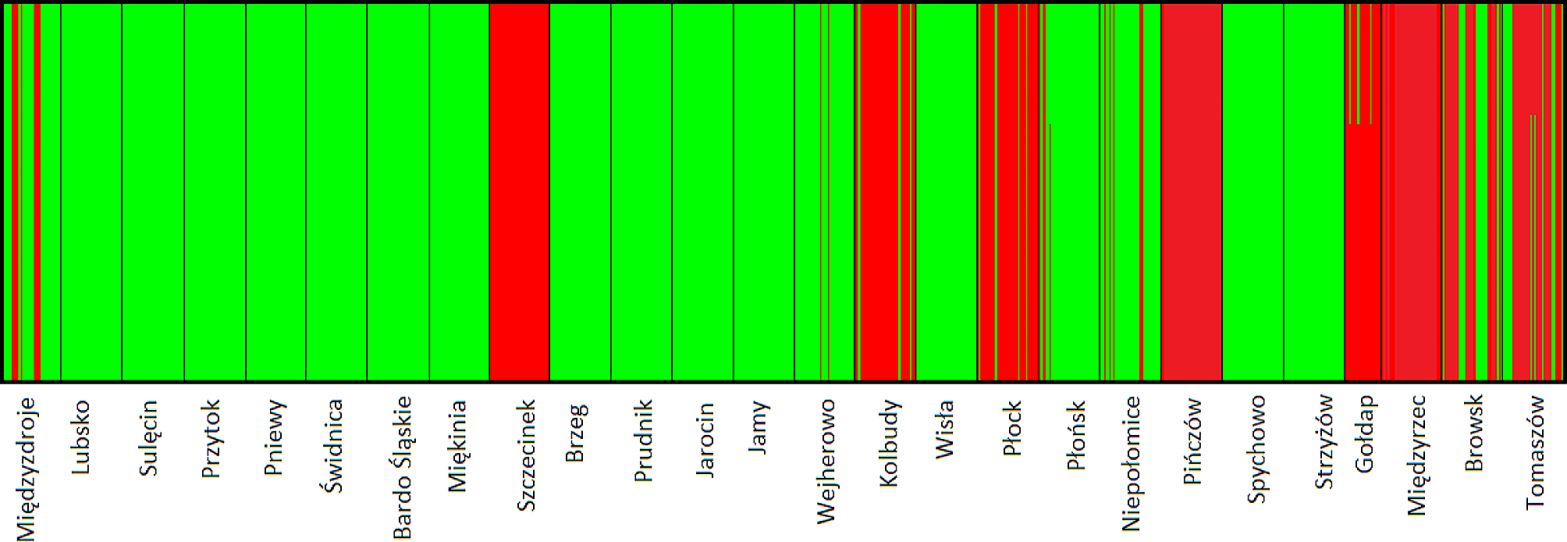
Genetic population structure of common ash for *K* = 2 in BAPS. Each individual is represented by a vertical line, while populations are separated by black lines

The Bayesian assignment test with BAPS identified *K* = 2 as the most propable number of genetic cluster (Fig. 2). For *K* = 2, the southwestern populations clustered into one group, and the northeastern populations clustered into the other group, although lineage admixture was observed in a few populations, where the distribution of the two lineages overlapped (Figs. 1c and 2c). Admixture levels (*D*) estimated based on individual assignment probabilities ranged from zero (17 populations) to 0.585 (Browsk), with the mean equal to 0.153 (Table S2).

The PCoA-based inference about genetic structure indicated three groups (Figure S2). The first group consisted of 72.2% of all individuals and corresponded to the first genetic group identified withBAPS. The second and the third group consisted of individuals assigned to the second genetic group identified with BAPS. Within the second group, PCoA included 81 individuals from the Międzyrzec, Płońsk, Strzyżów, and Tomaszów populations. All of these individuals possessed the H2 haplotype. The remaining individuals belonged to the third group.

#### Phylogeographic structure

Two haplotype groups showed a non-random geographic distribution (Fig. 1b). Additionally, the geographical distribution of two gene pools identified by BAPS (Fig. 1c) reflected the distribution of haplotype lineages (Fig. 1a). Generally, one gene pool (haplotypes *H1*, *H3*, *H5*) predominated in populations located in southwestern Poland, whereas the other (haplotypes *H2*, *H4*, *H6*) prevailed in populations located in northeastern Poland. The largest discrepancies were observed in populations located along the borderline of the two gene pools. The Mantel test indicated a significant relationship between genetic distance and geographic distance among populations (*r* = 0.118, *p* = 0.033).

## Nuclear microsatellites

### Genetic diversity

**Fig. 3:**
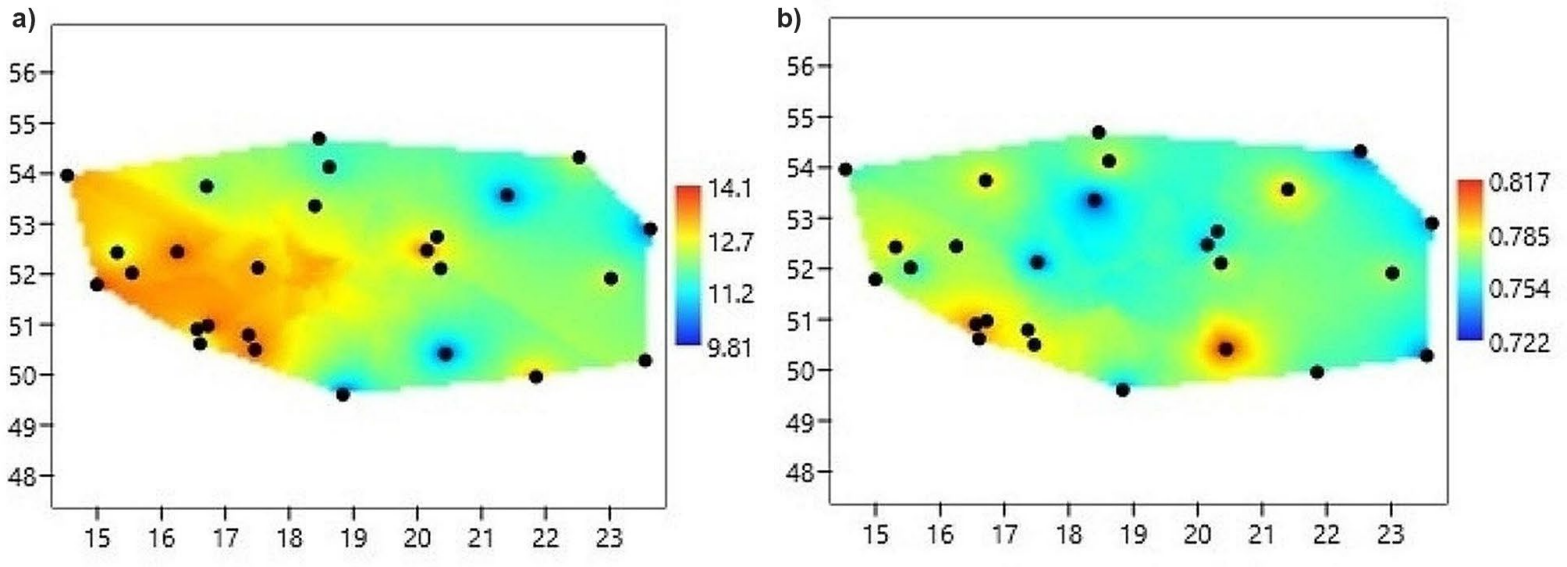
The spatial interpolation of (a) allelic richness and (b) the expected heterozygosity of the common ash in Poland

Detailed results for nuclear microsatellite loci are shown in Table S3. The average number of alleles within populations ranged from 10.9 (Spychowo) to 17.9 (Miękinia), with a mean of 14.5. The effective number of alleles was 7.38. Allelic richness measured after rarefaction ranged from 9.56 (Pińczów) to 14.92 (Miękinia). The mean observed heterozygosity (*H*_*o*_ = 0.542) was lower than the mean expected heterozygosity (*H*_*e*_ = 0.769). It is worth noting that genetic diversity based on the allelic richness and expected heterozygosity was the highest in western Poland (Fig. 3). Analyzed populations showed high values of the standard inbreeding coefficient (*F*_*IS*_), ranging from 0.195 (Pińczów) to 0.413 (Przytok), with a mean of 0.318. Generally, the genotype frequencies at the population level departed from Hardy-Weinberg expectations. Inbreeding coefficients corrected for null allele presence (*F*_*IS*_*INEST*) were noticeably lower than the standard *F*_*IS*_ and spanned between 0.014 and 0.173 (with a mean of 0.069; Table S4). Moreover, based on the model comparison, *F*_*IS*_*INEST* was an important (non-zero) component of the model in six populations only, indicating that the deficiency of heterozygotes was mostly due to null alleles, likely present in high frequencies at two loci, i.e. *3.15* (0.310) and *ASH53476* (0.273) (Table S3).

**Fig. 4:**
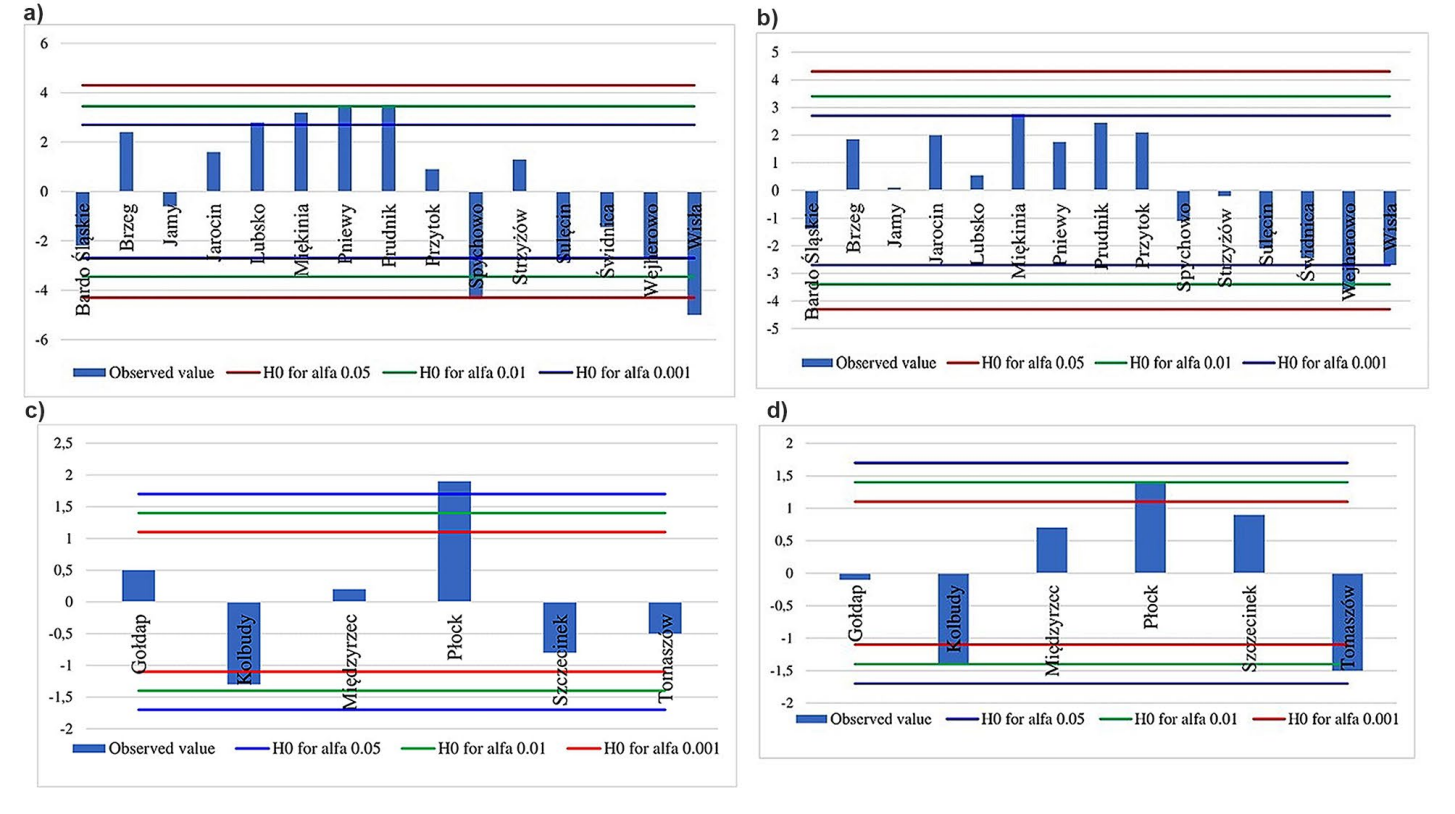
Heterogeneity of genetic variation levels within phylogenetic gene pools *Q*_*1*_ (a, b) and *Q*_*2*_ (c, d) in terms of allelic richness (a, c) and expected heterozygosity (b, d). The bars show the (centered) mean ranks for populations, while the lines indicate the bootstrap confidence intervals (for different levels of significance) around the null hypothesis (no deviation from the mean value)

As revealed by the Friedman test, genetic diversity parameters did not differ between phylogenetic gene pools (allelic richness: χ2 = 1.60, df = 1, *p* = 0.206; expected heterozygosity: χ2 = 0.40, df = 1, *p* = 0.527). However, both allelic richness and the expected heterozygosity were not homogeneously distributed among populations within phylogenetic gene pools (allelic richness for *Q*_*1*_: χ2 = 59.53, df = 14, *p* < 0.00001; the expected heterozygosity for first phylogenetic gene pool *Q*_*1*_: χ2 = 31.32, df = 14, *p* = 0.005; allelic richness for second phylogenetic gene pool *Q*_*2*_: χ 2 = 18.51, df = 5, *p* = 0.002; expected heterozygosity for *Q*_*2*_: χ 2 = 21.37, df = 5, *p* = 0.001). Within the first phylogenetic gene pool, four populations (Lubsko, Miękinia, Pniewy, and Prudnik) with the highest allelic richness were classified as positively outlying populations in terms of genetic polymorphism (Fig. 4a). Similarly, Płock was identified as a positive outlier within the second phylogenetic gene pool (Fig. 4c, d). On the other hand, four populations (Spychowo, Sulęcin, Wejherowo, and Wisła), with the lowest allelic richness or heterozygosity excess, were identified as negatively outlying populations in the first phylogenetic gene pool (Fig. 4a, b). In addition, Kolbudy and Tomaszów populations were identified as negative outliers in terms of both allelic richness and heterozygosity excess within the second phylogenetic gene pool (Fig. 4c, d).

### Genetic structure

The overall genetic differentiation was low but statistically significant, with *F*_*ST*_ = 0.045 (SE = 0.001) and *R*_*ST*_ = 0.057 (SE = 0.007). AMOVA showed that the majority of molecular variance was attributed to the within-population variation (73.24%), while the variation among populations was only 4.62%. In the case of STRUCTURE analysis of population genetic structure, the log probability of data did not allow us to infer unambiguously the best *K* value because the log probability tended to increase continuously with *K* (Figure S3a). On the other hand, the highest Delta *K* value was observed for *K* = 2, although the Delta *K* statistic [27] showed also additional peaks for *K* = 5 and 10 (Figure S3b), suggesting a hierarchical genetic structure (see Figure S3c). All individuals and locations showed high admixture levels. In addition, the assignments of samples to genetic groups did not reveal any clear geographic structure. The lack of geographic structure was confirmed by the Mantel test, which revealed positive but not significant correlation between geographic and genetic distances (*r*^2^ = 0.019, *p* = 0.161).

**Fig. 5:**
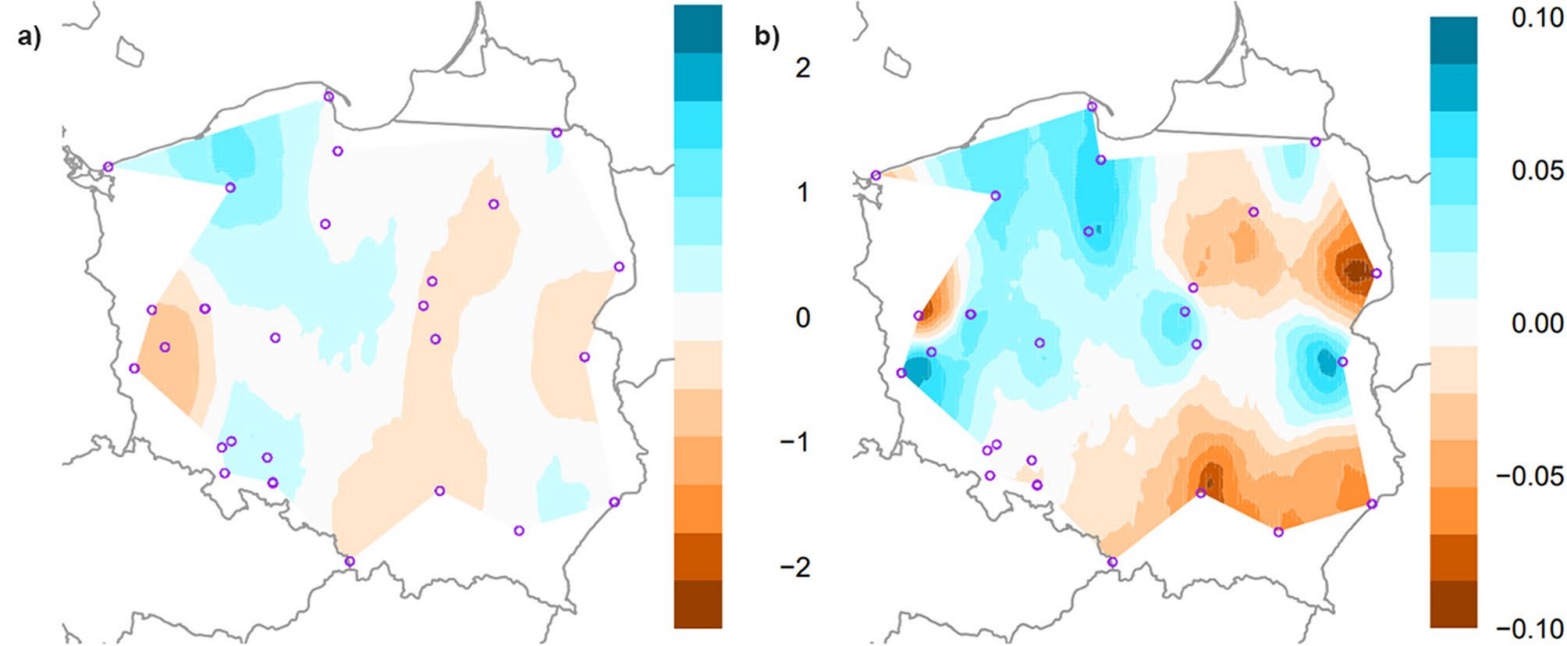
The estimated effective migration surface for *Fraxinus excelsior* in Poland: (a) the effective migration rates among populations and (b) diversity rates on a *log*10 scale

The results of estimated effective migration surfaces indicated less gene flow in the eastern part of Poland (Fig. 5). The largest zone of low genetic connectivity spanned more or less along the Wisła River (Fig. 5a). Whereas, in northwestern Poland, genetic diversity and gene flow were higher (Fig. 5b).

### Within-population spatial genetic structure

We observed a high variation of spatial genetic structure patterns between populations, with a significant spatial autocorrelation in 14 out of 23 populations (Table S5). Regression slopes (*b*_*log*_) were negative for all populations, except Pinczów, Świdnica, and Wejherowo, indicating that, on average, neighboring individuals tend to be more genetically related than individuals separated by a larger distance (Table S5, Figure S4). Kinship coefficients for the first distance class (*F1*) showed some variation among sites, with the maximum value of *F1* = 0.068 in population Jamy (Table S5). The neighborhood size (*N*_*b*_) estimated based on the *b*_*log*_ values varied from 51.52 (Gołdap) to 3594.36 (Pińczów) individuals, with the (harmonic) mean of 189.8 (Table S5).

### Demography

Estimates of contemporary effective population size (*N*_*e*_) ranged from 17.1 (Browsk) to *undefined* (theoretically *infinite*) (Strzyżów), with a harmonic mean of 57.5 (Table S6). None of the study populations showed the heterozygosity excess beyond the drift-mutation equilibrium (Table 1), suggesting the lack of recent genetic bottleneck in study populations. The observed *M*-ratios ranged from 0.504 (Spychowo) to 0.660 (Płock), indicating the deficiency of alleles in the study populations relative to the polymorphism expected for the observed allelic size range under the long-term demographic equilibrium (Table 1). Furthermore, all *M*-ratio values were lower than the critical value of 0.68 proposed by Garza and Williamson [28]. Thus, in opposition to the heterozygosity excess tests, the *M*-ratio tests suggested that historical bottlenecks had occurred in 23 out of 26 populations.

**Table 1:** Genetic bottleneck using heterozygosity excess and *M*-ratio tests

Population ID	Population	Heterozygosity excess			M-ratio		
		*H*_*e*_	*H*_*eq*_ (SE)	p	*M*	*M*_*eq*_ (SE)	p
1	Bardo Śląskie	0.739	0.788 (0.058)	1	0.605	0.766 (0.175)	< 0.0001*
2	Browsk	0.718	0.756 (0.062)	1	0.543	0.776 (0.178)	< 0.0001*
3	Brzeg	0.792	0.850 (0.041)	1	0.633	0.748 (0.167)	0.03*
4	Gołdap	0.772	0.828 (0.047)	1	0.519	0.717 (0.178)	< 0.0001*
5	Jamy	0.756	0.822 (0.048)	1	0.532	0.743 (0.174)	< 0.0001*
6	Jarocin	0.776	0.840 (0.044)	1	0.599	0.751 (0.169	0.01*
7	Kolbudy	0.754	0.829 (0.047)	1	0.529	0.760 (0.171)	< 0.0001*
8	Lubsko	0.784	0.851 (0.040)	1	0.629	0.741 (0.166)	0.04*
9	Międzyrzec	0.812	0.828 (0.047)	0.88	0.572	0.754 (0.171)	< 0.0001*
10	Międzyzdroje	0.805	0.854 (0.039)	1	0.603	0.751 (0.167)	< 0.0001*
11	Miękinia	0.794	0.838 (0.045)	1	0.627	0.737 (0.166)	0.05
12	Niepołomice	0.800	0.805 (0.055)	0.85	0.581	0.761 (0.173)	< 0.0001*
13	Pińczów	0.729	0.770 (0.064)	1	0.509	0.778 (0.179)	< 0.0001*
14	Płock	0.825	0.869 (0.034)	1	0.66	0.737 (0.164)	0.11
15	Płońsk	0.782	0.842 (0.042)	1	0.601	0.755 (0.169)	< 0.0001*
16	Pniewy	0.781	0.850 (0.041)	1	0.6	0.739 (0.167)	0.01*
17	Prudnik	0.801	0.859 (0.038)	1	0.614	0.742 (0.166)	0.01*
18	Przytok	0.807	0.828 (0.048)	0.98	0.628	0.749 (0.169)	0.02*
19	Spychowo	0.768	0.753 (0.063)	0.47	0.504	0.780 (0.178)	< 0.0001*
20	Strzyżów	0.731	0.839 (0.045)	1	0.659	0.744 (0.168)	0.11
21	Sulęcin	0.726	0.814 (0.052)	1	0.606	0.764 (0.173)	< 0.0001*
22	Szczecinek	0.831	0.807 (0.054)	0.81	0.573	0.764 (0.173)	< 0.0001*
23	Świdnica	0.726	0.820 (0.050)	1	0.549	0.759 (0.172)	< 0.0001*
24	Tomaszów	0.750	0.829 (0.047)	1	0.586	0.752 (0.172)	< 0.0001*
25	Wejherowo	0.724	0.839 (0.044)	1	0.573	0.759 (0.170)	< 0.0001*
26	Wisła	0.711	0.739 (0.068)	1	0.519	0.778 (0.180)	< 0.0001*

*H*_*e*_ – expected heterozygosity, *H*_*eq*_ - expected heterozygosity at mutation-drift equilibrium, *M* - *M*-ratio, *M*_*eq*_ - *M*-ratio at mutation-drift equilibrium, *statistical significance of *p* < 0.05

### Patterns of genetic diversity and structure

The genetic parameters estimated for the study populations showed significant geographic trends. The number of alleles and the historical bottleneck index were significantly correlated with longitude (*r* = -0.41; *p* < 0.05 and *r* = -0.45; *p* < 0.05, respectively), indicating that populations located farther east have a lower number of alleles and a higher genetic bottleneck signal. In contrast, no significant correlation was observed between genetic parameters and phylogenetic affiliation. However, the level of phylogenetic admixture was negatively correlated with the inbreeding coefficient (*r* = -0.68, *p* < 0.05). Finally, genetic parameters revealed significant associations with linkage disequilibrium caused by (the recent) genetic drift (Table 2).

**Table 2:** Spearman’s correlation coefficients for all variables in the study

Parameter	Longitude	Latitude	Q_1_	D	r^2^|Drift
*A*	– 0.41*	– 0.21	0.26	– 0.08	-0.78***
*A*_*e*_	– 0.34	– 0.09	0.26	– 0.20	-0.58**
*A*_*R*_	– 0.34	– 0.11	0.21	– 0.11	-0.71***
*F*_*IS*_ *INEST*	-0.17	-0.04	0.11	-0.68*	-
*Sp*	0.05	0.04	-0.03	-0.35	-
*r* ^*2*^ *|Drift*	0.26	0.25	-0.27	0.40	-
*H*	0.19	0.04	-0.19	0.06	0.67***
*M*	-0.45*	-0.3	0.29	-0.01	0.31

*A* – number of alleles, *A*_*e*_ – effective number of alleles, *A*_R_ – allelic richness, *F*_*IS*_*INEST –* inbreeding coefficient, *Sp – the* intensity of SGS, *r*^*2*^*|Drift –* linkage disequilibrium with sample correction, *H –* recent bottleneck (*H =* H_e_ - H_eq_ ), *M –* historical bottleneck, *Q*_*1*_*–* population affiliation to the first phylogenetic lineage, *D* – phylogenetic admixture, statistical significance:***p < 0.001; **p < 0.01; *p < 0.05, n.s. – not significant

## Discussion

In this study, we characterized the genetic diversity and population genetic structure of common ash in Poland, making the first step towards the delineation of seed transfer zones as well as a genetic basis for the restoration and conservation of the species. The detailed results of this work are discussed in the appropriate thematic sections.

### Phylogenetic structure

Chloroplast microsatellite markers revealed a low level of genetic diversity, with only six haplotypes at three polymorphic loci. Differentiation among populations was high, with 69.5% of molecular variance attributed to differences between populations. Differentiation in ash was higher than in seven other species with samara seeds (average *G*_*ST*_ = 0.660 [29]). The study at a local spatial scale provided arguments that seeds of common ash disperse over relatively short distances [30], causing a poor mixing of maternal lineages.

Phylogenetic relationships among haplotypes, as well as the analysis of genetic structure, revealed the existence of two gene pools. The first haplotype group comprised populations located mostly in southwestern Poland. The second group tended to prevail in the northeast of the country. Based on the distribution of cp.DNA types presented in Heuertz et al. [17], the migration of common ash trees towards the north occurred through two distinct migration paths that probably met in northwestern region of Poland. A more recent study [31] confirmed the existence of two evolutionary lineages of ash in Poland, indicating that the Central European lineage of common ash dominates in Poland, with a share of about 70% of the studied populations. The analysis of pollen diagrams from the northern Alps and the Carpathians reveals that there were significant population expansions of common ash trees during the early Holocene, suggesting that the glacial distribution of this species was large and might have included regions located more northwards such as the Carpathian Basin [32]. This may explain the dominance of two evolutionary lineages in Central Europe. Pollen diagrams from southeastern Europe indicated a late presence of common ash in the Balkan Peninsula [33, 34]. Earlier occurrence of the Central European lineage of common ash in Poland might have constrained the spreading of the eastern gene pool in the southwest direction.

The population differentiation in cp.DNA increased significantly with the logarithm of geographical distance, although the regression line explained only 14% of the total genetic differentiation. A similar result was obtained at the Pan-European scale, where the pattern of isolation by distance explained up to 10% of the total genetic variation. The isolation by distance is shaped by the balance between genetic drift and gene flow, so the observed positive correlation between genetic and geographical distance can result from limited gene dispersal and low population density. In ash, high *F*_*ST*_ values and a relatively low level of isolation by distance suggest a dominant role of genetic drift over gene flow due to seed dispersal, which may drive founder events.

### Genetic diversity and differentiation

Genetic diversity of common ash based on nuclear microsatellite markers (*H*_*e*_ = 0.769) was close to the average level in other parts of the species natural range (*H*_*e*_ = 0.770, [18]). Only, the population in Italy had a higher level of genetic diversity (*H*_*e*_ = 0.840, [35]). However, the observed genetic differentiation among populations in Poland was low (*F*_*ST*_ = 0.049) and explained only 4.6% of the total molecular variance among populations, as revealed by the AMOVA. In comparison, the average estimate of population differentiation among 322 woody taxa for allozyme loci was *G*_*ST*_ = 0.084 [36], whereas lower estimates were obtained for oaks (*G*_*ST*_ = 0.024 and 0.032 in *Quercus petraea* and *Q. robur*, respectively [37] and beech (*Fagus sylvatica*, *G*_*ST*_ = 0.054, [38]; *F*_*ST*_ = 0.046, [39]). The overall pattern of genetic diversity and differentiation between populations at nuclear microsatellite loci observed for the common ash is typical for a long-lived outcrossing species [36].

There was neither a significant pattern of isolation by distance nor geographic grouping of populations, as revealed by the UPGMA dendrogram. This is undoubtedly due to extensive gene flow through pollen between populations in the study species [25, 40]. In this regard, our study contrasts with the earlier studies. The among-population IBD pattern was found in Bulgaria (r^2^ = 0.152, *p* ≤ 0.001; [41] ), western and central Europe (r^2^ = 0.190, *p* ≤ 0.001; [18]) and in southeastern Europe (r^2^ = 0.090, *p* ≤ 0.01; [18]). The significant IBD patterns within south-eastern Europe could indicate the colonization of the area from various glacial refuges [18]. Interestingly, the absence of a significant IBD pattern at the continental scale can be attributed to the strong differentiation observed over relatively short distances in southeastern Europe, which masked the spatial genetic pattern characterized by a weaker differentiation among populations in western and central Europe [18].

Genetic clustering revealed two major genetic groups (see Figure S3). However, individual assignments to gene pools showed no geographical structure, confirming the results obtained at the among-population level. In addition, although the models for *K* > 2 had lower statistical support, our results suggest the existence of local genetic structure, where populations are characterized by the predominance of one genetic lineage. The local genetic structure is probably related to the way the populations have been established, such as the scenario with bottleneck events followed by the among-population pollen-mediated gene flow [42]. The establishment of such populations is usually associated with a limited number of seed sources or seed dispersal from a single location. On the other hand, the regional structure of common ash could be associated with adaptation to the local conditions. However, Clark [43] found no evidence of local adaptation of common ash populations in France and England, which is also confirmed by previous studies [44].

### Geographic structure and inbreeding levels

In contrast to the among-population scale, there was a significant spatial genetic structure within ash populations. A significant linear decline in estimated kinship coefficients was observed as the logarithm of geographical distance increased. However, the decay of kinship was not uniformly linear over the whole distance range, with a steeper kinship decrease at a short distances, and a shallower decrease at large distances (Figure S4). As revealed by the simulations, the observed pattern can be attributed to restricted seed dispersal as compared to pollen dispersal [30]. The restricted seed dispersal may reflect the high vertical terminal velocities of ash seeds (1.2–1.7 m/s, [45, 46]) dispersing under a relatively closed forest canopy where wind velocity is reduced [47]. Heuertz et al. [30] also found a steep decrease of kinship at short distances in ash populations in Romania, which is expected when gene dispersal follows a highly leptokurtic distribution. The scenario described above arises when the dispersal of seeds is considerably lower than that of pollen. This is because half of the genetic material, specifically the maternal genes, tends to move over shorter distances, while the other half, the paternal genes, can disperse over longer distances. Consequently, this process leads to a composite distribution with a leptokurtic shapeInterestingly, we did not observe any evidence of spatial genetic structure in 9 out of the total 23 populations. This could be due to the specific population history, i.e. a recent origin or forest management [48]. Alternatively, it could be a result of the sampling area being smaller than the actual neighboring area within these forest stands. The average strength of spatial genetic structuring expressed by the Wright’s neighborhood size of *N*_*b*_ = 190 was lower than the value obtained for the common ash in Romania (*N*_*b*_ = 519 [30]), indicating a stronger within-population spatial genetic structure in norther ash populations, likely due to a more scattered (or fragmented) distribution of the species in the northern part of the range. A similar trend has been found in leading-edge populations of *Acer campestre* in Poland, which also display the increase in the rate of population divergence along with latitude [49]. More data are needed, however, to get robust insights in this respect.

The study populations were characterized by a significant deviation from the Hardy–Weinberg equilibrium, generated by the heterozygote deficiency. Possible causes behind the observed pattern include null alleles, self-fertilization, biparental inbreeding [30], as well as the Wahlund effect [50]. Relatively high proportions of null alleles were estimated for the microsatellite loci, except for *FE11* and *ASH7867* (Table S3). Similar results regarding null alleles were noted by Morand et al. [51] for *FE11* and *FE19* loci in France, as well as Hebel et al. [52] for *FE12* and *FE16* loci in Germany. As most of the loci showed the presence of null alleles, we calculated also *F*_*IS*_ using a method robust to the presence of null alleles which revealed that the inbreeding coefficient was significantly different from zero only in 6 populations. Therefore, the observed heterozygote deficiency can be attributed to the presence of null alleles. Consequently, despite the trend towards clustering of related individuals within populations, which may lead to mating between relatives, only weak signs of inbreeding were observed in ash populations in Poland. Except for the two populations (Spychowo and Tomaszów), the mean inbreeding coefficient (*F*_*IS*_ = 0.077) was higher than the average kinship coefficient between neighbor trees (*F* = 0.022), suggesting that the observed inbreeding was mostly due to self-fertilization. However, our results contrast somewhat with those observed in Bulgaria, where the average values of inbreeding coefficient never exceeded kinship coefficients between neighbor trees, suggesting that inbreeding was generated by mating between related individuals whereas the contribution of selfing was negligible [41].

It should be stressed that trees often reveal dioecy in the common ash [35]. However, individual trees can reveal a spectrum of sex morphs, ranging from pure males to pure females, with a variety of hermaphroditic intermediates in between [53, 54]. Therefore, selfing in the species is somewhat unexpected, as confirmed by direct estimates based on progeny arrays [25]. Because our estimates accounted for null alleles while the Wahlund effect could be excluded (based on the STRUCTURE results), the assortative mating between closely related but not necessarily spatially close individuals remains a possible explanation. However, additional data on morphology, phenology, and sexual phenotype within populations are necessary to resolve causes of excessive inbreeding observed incidentally in ash populations.

### Patterns of genetic diversity

The genetic diversity tended to decrease with longitude (Table 2). Assuming that the colonization wave after the last glacial maximum (LGM) proceeded toward the East, such a genetic pattern appears to be consistent with the leading-edge colonization model [55]. Long-distance dispersal events likely played a significant role in overcoming the physical barriers [41], but their frequency was insufficient to counterbalance the loss of genetic diversity caused by genetic drift resulting from the founder effect. The evidence of founder events in recently recolonized ash populations was strong because signs of historical bottleneck were detected in almost all populations (the *M*-ratio test, Table 1.), despite the high genetic diversity. The balance between the number of alleles and heterozygosity indicates that demographic events leading to the reduction of genetic variation occurred long ago in a population history and can be related to the colonization after LGM.

According to the central-marginal hypothesis, the reduction in genetic diversity observed in the peripheral areas of a species’ range can be attributed to a smaller effective population size and increased geographical isolation when compared to populations located in the central regions [56–59]. Eckert et al. [60] sought to quantify the extent to which the central-peripheral hypothesis is supported by data. They showed that genetic diversity declined towards the range margins in 64.3% of the analyzed studies. Nevertheless, in the majority of cases, the disparity in genetic diversity between central and peripheral populations was small. Additionally, very few studies have incorporated a phylogeographic framework to assess the historical influences on the contemporary genetic pattern [61, 62]. In our study, the pronounced loss of genetic diversity with increasing distance from the refuge areas was observed. When set into a phylogeographic context, our results indicated that the postglacial history remains the central determinant of the genetic structure of common ash in Poland.

### Demographic history

As a measure of genetic drift, the effective population size (*N*_*e*_) is directly related to the rate of loss of genetic diversity and the rate of increase in inbreeding within a population [63]. The decrease in genetic variation can impact population dynamics and long-term survival through three mechanisms: inbreeding depression, the loss of phenotypic variation, and the loss of evolutionary potential [64]. Maintaining populations large enough to minimize such effects has become a crucial objective in the management of common ash threatened by *Hymenoscyphus fraxineus*. The effective population size was relatively high for the majority of populations, indicating that many individuals participate in the transmission of genetic variation to the next generation. However, 8 out of 26 populations (Browsk, Gołdap, Międzyzdroje, Pińczów, Spychowo, Sulęcin, Tomaszów, and Wisła) had a low effective population size (below 50), being under the risk of genetic erosion if the *N*_*e*_ continues to be low in future generations. Although the effective population size might occasionally decline below 50 without negative consequences, the maintenance of adaptive genetic variation over longer periods (e.g., centuries) requires *N*_*e*_ of more than 500 individuals [64]. With the current knowledge, it remains unclear whether the long-distance pollen dispersal, observed in the study species [25, 40], will suffice to counter-balance the local genetic drift, especially taking into account decreased fecundity due to ash dieback. Therefore, genetic monitoring of progeny is required for assessing whether local genetic diversity reveals any worrisome symptoms.

In our study, no population displayed a significant heterozygosity excess, suggesting that all populations have not experienced a recent genetic bottleneck. In contrast, the signs of historical bottleneck were observed in almost all populations due to the disproportion between the number of alleles and their size range (*M*-ratio). Probably, historical bottlenecks may be associated with the postglacial recolonization of common ash. We found that the historical bottleneck intensity increased with longitude. In addition, the number of alleles decreased with increasing distance from the refugium. The recolonization of common ash in Poland started quite late after the LGM (around 7,000 years ago), when other tree species had already re-colonized suitable sites [65]. Colonization into already occupied areas might have had a stronger selection effect compared to the selection after colonization of open lands and might have produced strong founder effects leading to the loss of variation. In our study, the haplotype *H6* remained “trapped” in a relatively small area, and none of the rare haplotypes (except *H4*) was observed in more than one or two populations. Nevertheless, the evidence of founder events in recently recolonized ash populations in Europe is generally weak, because gene diversity is high and bottleneck indices remain mostly non-significant [18]. Non-significant heterozygote excess tests in the studied populations suggest that the observed genetic pattern may be explained through the recent admixture of genetic lineages as a result of merging postglacial recolonization routes [29], as well as a recent intense gene flow [30].

### Management and conservation implications for *Fraxinus excelsior* in Poland

Delineation of areas within which seeds (or seedlings) can be transferred with little maladaptation risk, often called seed zones or provenance regions, is a common forestry practice [10, 66, 67]. A pivotal assumption is that local populations have higher fitness (i.e. survival, reproduction, disease resistance, or abiotic resilience) than non-local populations [9, 68]. By utilizing locally adapted populations, not only are alleles that are well-suited to local conditions preserved, but it also helps avoid the introduction of genotypes that are ill-suited or maladapted to the specific environment [68, 69]. A reliable procedure to delineate seed zones should be based on robust genetic data. Chloroplast marker variation revealed two evolutionary lineages of common ash in Poland. A geographical distribution of chloroplast DNA diversity as compared with the results obtained by Heuertz et al. [17] indicates, that the first phylogenetic lineage covering populations from southwestern Poland is assigned to the refugium from the eastern Alps, while the second phylogenetic group including populations from the northeast of the country belonged to the refugium from the Balkan refugium. Any replacement of native trees for whatever reason should be based on the knowledge of the geographic distribution of genetic variation [70]. Our results suggest that ash stands across Poland can be treated as two management units. Therefore, it is not recommended to transfer reproductive material between the southwestern and the northeastern part of the country.

Preservation of the possibly wide genetic variation of common ash is of fundamental importance for the stability of forest stands and whole forest ecosystems. Generally, ash stands in Poland are characterized by a relatively high level of genetic variation, expressed by the allelic richness and the expected heterozygosity (Table S4, Fig. 3). Genetic diversity parameters were similar in the two phylogenetic gene pools. However, the Friedman test detected significant differences in genetic diversity across populations within phylogenetic gene pools. Our results identified several common ash populations as a rich source of genetic variation within each phylogenetic gene pool indicating a high conservation value of these populations for this species. (Fig. 4). In periods of climate change and ash dieback, ash populations face strong selective pressures [21, 22]. The invasive pathogenic fungus *Hymenoscyphus fraxineus* has already caused severe damage in natural common ash populations across Europe during the last two decades, diminishing standing genetic variation. Only a limited number of trees show resistance against the pathogen [71]. Under these circumstances, genetically diverse populations might be important for the conservation of genetic resources and the evolutionary potential of this species. On the other hand, gene pools of six populations (Kolbudy, Spychowo, Sulęcin, Tomaszów, Wejherowo, and Wisła) may need to be enriched by genetic reproductive material from the most genetically variable populations. However, such actions must take into account the observed genetic structure of the species.

On a larger geographic scale, seed transfer may be particularly important in northeastern Poland, where populations showed a relatively low allele number (Table 2) and multiple signs of the bottleneck (Table 1). The historical reduction of population size is probably associated with the recolonization of ash in Poland. However, due to the possibility of new demographic events resulting from the destructive effects of *Hymenoscyphus fraxineus*, there is a real threat of genetic erosion, which could change the observed patterns of genetic structure. Therefore, it seems reasonable to monitor the genetic diversity of common ash populations in Poland.

In the future, it seems necessary to determine the genetic variation of common ash with respect to resistance to *Hymenoscyphus fraxineus*. The presence of natural resistance offers the potential to sustain the species by effectively managing the available natural resources [72]. In recent studies, Semizer-Cuming et al. [25, 73] found a positive correlation between resistance to ash dieback and reproductive success. In fact, trees resistant to ash dieback produce more seeds than susceptible trees [25], which gives hope for the preservation of the species. On the other hand, the selection and propagation of highly resistant ash trees, followed by the restocking of forests, offers a potential route to revitalize and restore ash forests.

## Conclusions

Our results revealed the presence of two phylogenetic lineages of common ash in Poland. Both gene pools were characterized by equally high genetic diversity at nuclear markers, suggesting a similar evolutionary potential across the species range in Poland. The low differentiation between gene pools at the nuclear genome level and also the high level of admixture indicate intensive pollen gene flow in the species, which blurs the phylogenetic structure observed at the chloroplast genome level. It seems that such high pollen gene flow between populations is enough to maintain the genetic diversity of the species. However, it is essential to note that significant variation in genetic diversity among populations within phylogenetic lineages can primarily be attributed to demographic events. While the presence of *H. fraxineus* may pose challenges to preserving seed zones, given the relative abundance of ash populations in both major genetic groups, it is advisable to transfer reproductive material within the areas of homogeneous phylogenetic origin. However, further studies are needed to identify links between genetic diversity and resistance to the pathogen and the overall adaptive potential. A promising path is the application of next-generation sequencing (NGS) to study associations between genetic diversity and ash dieback damage status to find the most resistant ash genotypes to *H. fraxineus.* The availability of planting stock enriched with resistance genotypes seems to be necessary for the restoration of common ash. In the meantime, current conservation efforts should involve genetic enrichment of those populations which showed up as negative “genetic diversity” outliers. Such practices, however, need to be restricted to the within-lineage seed or seedling transfer.

## Materials and methods

### Plant material

Twenty-six populations of common ash (*Fraxinus excelsior* L.) were sampled within the NATURA 2000 sites network across Poland. The sampled populations are considered to be of natural origin. Leaf samples were collected from 30 to 51 adult trees per population at the turn of May and June 2015 (Table S7), yielding a final set of 1269 individuals. Collected samples were left to dry out at room temperature and stored in paper envelopes before DNA extraction.

### Molecular methods

Total genomic DNA was extracted from 20 mg of dried leaf tissue. The plant material was frozen and homogenized in a Mixer Mill MM301 (Retsch, Haan, Germany). DNA was extracted using the GeneMatrix Plant and Fungi DNA Purification Kit (EURx, Gdańsk, Poland), according to the manufacturer’s protocol. DNA amount and quality were evaluated using Biophotometer (Eppendorf, Hamburg, Germany). We assayed five chloroplast microsatellite markers (cp.SSR): *ccmp2*, *ccmp5*, *ccmp6*, *ccmp10* [74] and *µkk2* [75] and ten nuclear markers: *Femsatl4*, *Femsatl8*, *Femsatl11*, *Femsatl16*, *Femsatl19* [76], *Femsatl12* [77], *M2-30*, *3.15* [78], *ASH7867* and *ASH53476* [79]. Polymerase Chain Reaction (PCR) was performed in three multiplex reactions as described in the Supplementary Materials (Table S8). The fluorescently-labeled PCR products, along with the size standard the GeneScan 600 LIZ (LifeTechnologies Corporation, Carlsbad, CA, USA), were separated on a capillary sequencer ABI PRISM 3130XL® (Applied Biosystems, Foster City, CA, USA). The identification of alleles was performed using GENESCAN 3.7 and GENOTYPER 3.7 software provided by the manufacturer.

### Data analysis

#### Phylogenetic structure analysis

Haplotypes were determined as a combination of different microsatellite variants across the cp.SSR loci. The phylogenetic relationships of the established cp.SSR haplotypes were inferred based on the median-joining method [80]. The maximum parsimony analysis was conducted using the software NETWORK ver. 4.6.1.2 (Fluxus Technology Ltd, http://www.fluxusengineering.com). The chloroplast haplotype variation within populations (the total number of haplotypes, *N*, the effective number of haplotypes, *N*_*e*_, and genetic diversity, *GD*) was characterized using HAPLOTYPE ANALYSIS© ver. 1.05 software [81].

Genetic structuring at cp.DNA among and within populations was assessed using the analysis of molecular variance (AMOVA), implemented in ARLEQUIN version 3.0 (Excoffier [82], with significance tests based on 1,000 permutations. Parameters of genetic differentiation (*G*_*ST*_, *R*_*ST*_) were estimated using PermutCpSSR ver. 2.0 software [83], available at http://www.pierroton.inra.fr/genetics/labo/Software/PermutCpSSR/index.html. To investigate relationships between populations, the neighbor-joining (N-J) dendrogram [84] was constructed based on Nei’s *D*_*A*_ genetic distance [85] using Poptree Version 2 [86]. Statistical support for nodes was obtained based on 1,000 bootstraps over loci. In addition, individual samples and populations were assigned to different genetic groups using Bayesian assignment algorithm implemented in BAPS version 6.0 [87, 88] based on genotype data. The plausible number of clusters (*K*) was defined as a range between 1 and 25. The analysis was run with a burn-in period of 10,000 iterations followed by 50,000 iterations.The admixture level for each population was estimated using the Gini-Simpson coefficient following Chybicki et al. [49]. The genetic relationships between chloroplast individual genotypes were further investigated by principal coordinate analysis (PCoA) based on the PhiPT genetic distance matrix [89] in GenAlEx v.6 [90].The isolation by distance was tested using the Mantel test [91] of association between the *F*_*ST*_-based pairwise genetic distance matrix (i.e. *F*_*ij*_/ (1 – *F*_*ij*_), where *F*_*ij*_ is the *F*_*ST*_ for the i-th and the j-th populations) and the matrix of the natural logarithm of geographic distance using the GENEPOP version 4.4 software [92]. The significance was assessed with 10,000 random permutations.

#### Genetic diversity analysis

To characterize genetic diversity, nuclear microsatellite data were analyzed as follows. Each locus was tested for a deviation from the Hardy-Weinberg equilibrium using the exact test implemented in GENEPOP version 4.4 software [92]. Significance levels were adjusted using the sequential Bonferroni correction [93]. The genetic diversity within and among populations was estimated based on the following parameters: number of alleles (*A*), allelic richness (*A*_*R*_), observed heterozygosity (*H*_*o*_), and expected heterozygosity (*H*_*e*_) using FSTAT v 2.9.3 [94]. The effective number of alleles (*A*_*e*_) was calculated using GenAlEx 6 [90]. Differences in genetic diversity measures (i.e. *A*_*R*_, *H*_*e*_) between phylogenetic gene pools and populations within phylogenetic gene pools (population and gene pool as a factor) were tested with the Friedman rank test (Sheskin, 2000) implemented in BayeF [95]. Populations within phylogenetic gene pools were selected based on their affiliation to the phylogenetic lineage according to the criteria *Q* > 0.9. As a result, 15 and 6 populations were respectively assigned to the first and second phylogenetic gene pools. Positive and negative outliers were identified as the observations outside the limits of 95% confidence intervals determined using the bootstrap procedure (based on 1000 pseudo-samples).

The inbreeding coefficient (*F*_*IS*_) was estimated simultaneously with null allele frequencies using the Individual Inbreeding Model (IIM) implemented in the INEST 2.0 software [96]. The importance of inbreeding as an explanatory parameter was assessed by comparing the full model with the random mating model (i.e. when *F*_*IS*_ is 0) using the Deviance Information Criterion (DIC) implemented in INEST.

Differentiation among populations was measured using *F*_*ST*_ [97] and *R*_*ST*_ [98]. *F*_*ST*_ was estimated using the FreeNA package [99] with the ENA procedure to correct for the effect of null alleles. *R*_*ST*_ was calculated in SPAGeDI 1.3.d [100]. The hypothesis that *F*_*ST*_ = *R*_*ST*_ was tested using a permutation test (10,000 permutations) implemented in SPAGeDI 1.3.d.

To elucidate the genetic structure, we conducted Bayesian genetic clustering using STRUCTURE 2.3.4 [101, 102]. The number of tested genetic clusters (*K*) varied from 1 to 25, and five independent analyses were run for each *K*. The number of discarded iterations (burn-in) was set to 500,000, and the final run length was set to 1,000,000. The plausible number of clusters was inferred using the Structure Harvester software [103]. Additionaly, to assess genetic structure, the analysis of molecular variance (AMOVA), and the test of isolation by distance were performed as described earlier (see Phylogenetic structure analysis).Spatial genetic structure (SGS) was investigated as the relationship between pairwise kinship coefficients [104] and the spatial distance between individuals. Due to the lack of individual geographical coordinates in the Browsk, Jarocin, and Międzyzdroje populations, the SGS analysis was carried out in 23 populations. For each population, kinship coefficients were regressed against the log spatial distance between individuals (*d*_*ij*_) to compute the regression slope *b*_*log*_. The statistical significance of *b*_*log*_ was tested based on 10,000 permutations of spatial positions of individuals within a population. Standard errors of *b*_*log*_ were calculated by jackknifing over loci. To compare the strength of SGS between populations, the *Sp* statistic [105] was calculated as –*b*_*log*_/(1 – *F1*), where *F1* is the average kinship coefficient between individuals within the first distance class (automatically defined by the program). All calculations were performed using the SPAGeDI 1.3.d software [100].

Spatial variation in genetic diversity and gene flow rates were estimated using Estimated Effective Migration Surfaces (EEMS) [106]. We set the number of demes to 200 and ran five independent analyses with 1,000,000 burn-in Markov chain Monte Carlo steps and 4,000,000 iterations and a thinning interval of 9999. The results were visualized using rEEMSplots R package [106].

Estimates of effective population size (*N*_*e*_) were obtained per population using the linkage disequilibrium (LD) approach (the minimum allele frequency threshold of 0.02) implemented in LDNe v.1.0 [107]. Demographic population dynamics were evaluated using two different methods. The occurrence of a recent genetic bottleneck was evaluated by the heterozygosity excess test [108], while the historical bottlenecks were tested using the *M*-ratio test [28]. The significance of a bottleneck signal was computed using the Z test to compare observed (*H, M*) and the equilibrium values (*H*_*eq*_, *M*_*eq*_), where the equilibrium values were computed by coalescent simulations assuming the two-phase mutation model (TPM) using the default settings in the INEST 2.0 software [96].

### Patterns of genetic diversity and structure

To identify geographic trends in the estimated genetic characteristics, we calculated correlations between longitude and latitude, and the following parameters: *A, A*_*e*_, *A*_*R*_, *F*_*IS*_*INEST, Sp, r*^*2*^*|Drift, H*, and *M*. The phylogenetic trends were tested using correlations between population affiliation to the first phylogenetic lineage (*Q*_*1*_) and phylogenetic admixture (*D*), both computed based on chloroplast markers and parameters of genetic diversity and structure of the nuclear genome. Using the same method, the relationship between the inbreeding coefficient (*F*_*IS*_*INEST*) and spatial genetic structure (*Sp*) was tested. In addition, the relationship between linkage disequilibrium (*r*^*2*^*|Drift*) and genetic diversity parameters (*A, A*_*e*_, *A*_*R*_), as well as population demographic history (*H, M*) was determined. To account for potential non-linear relationships, the Spearman rank correlation coefficient was used (STATISTICA version 12.5).

### Electronic supplementary material

Below is the link to the electronic supplementary material.

Supplementary Material 1

Supplementary Material 2

## Data Availability

The dataset of individual genotypes with information about sampling sites is available on Zenodo: 10.5281/zenodo.5574905.
