# Supplementary material for: Genetic resources of common ash (Fraxinus excelsior L.) in Poland
Source: BMC Plant Biol. 2024 Mar 13;24:186. doi: 10.1186/s12870-024-04886-z (PMC10935948; doi:10.1186/s12870-024-04886-z)
Supplement: Supplementary file 1 — Supplementary Material 1 [file 12870_2024_4886_MOESM1_ESM.docx]

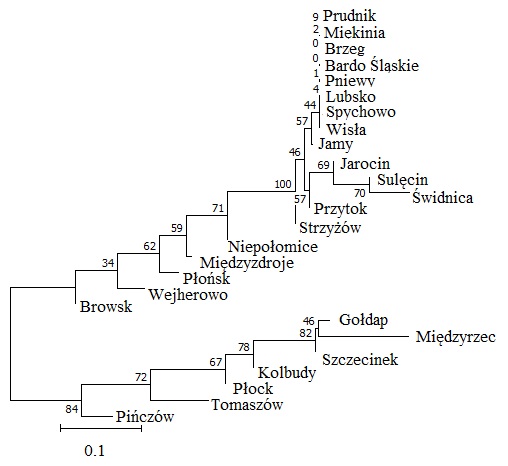


**Figure S1.** Neighbor-joining tree calculated using the Nei distance based on chloroplast microsatellite loci.


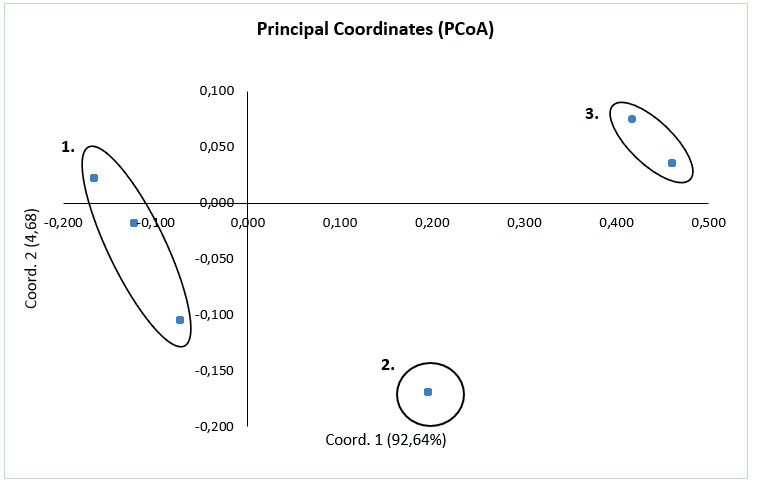


**Figure S2.** Principal coordinates analysis (PCoA) plot.

| a  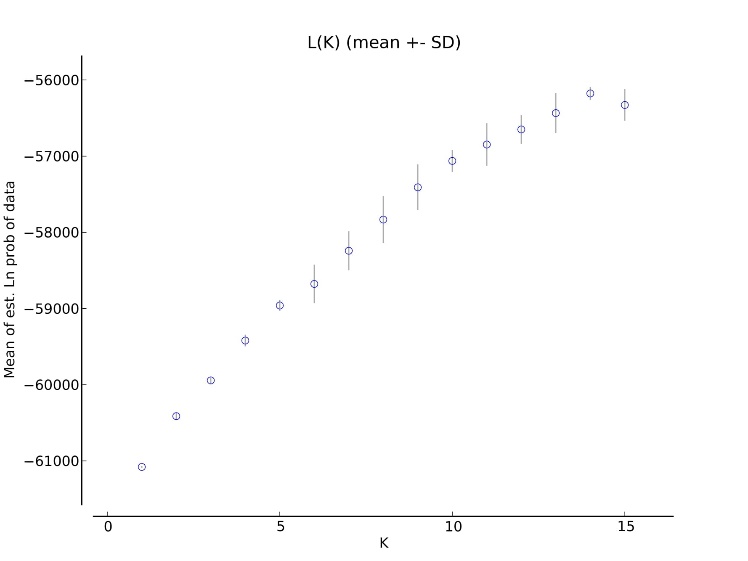 | | b  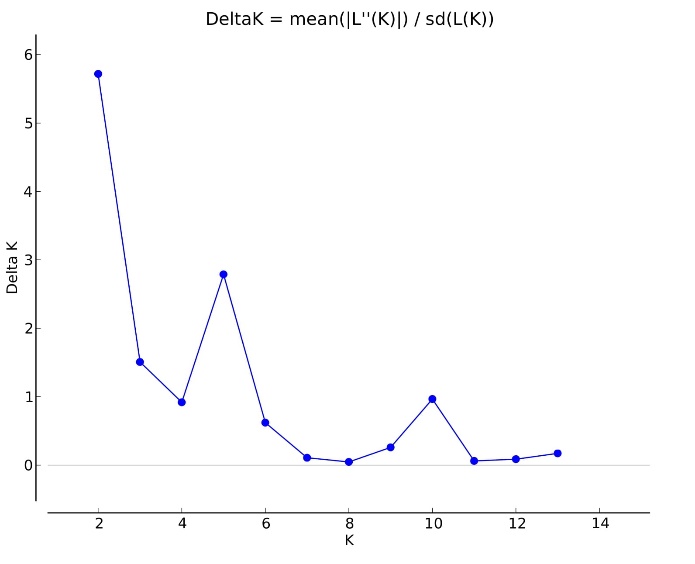 |
| --- | --- | --- |
| c  *K* = 2 | 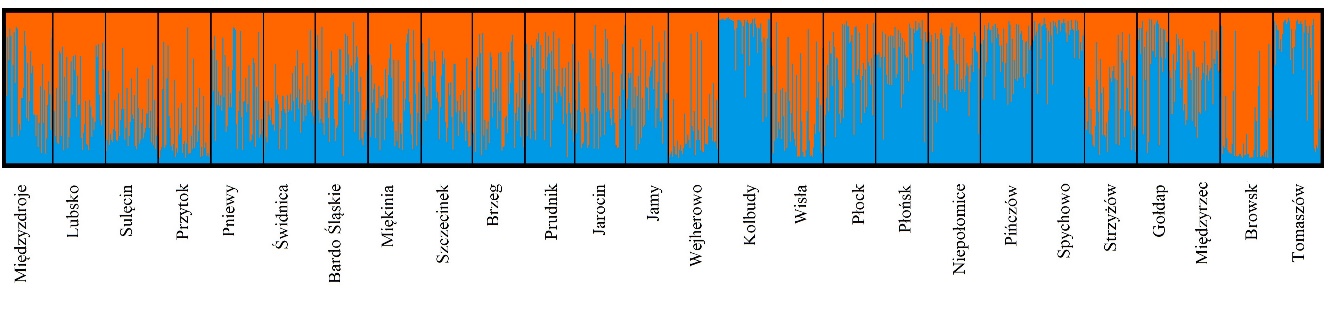 | |
| *K* = 5 | 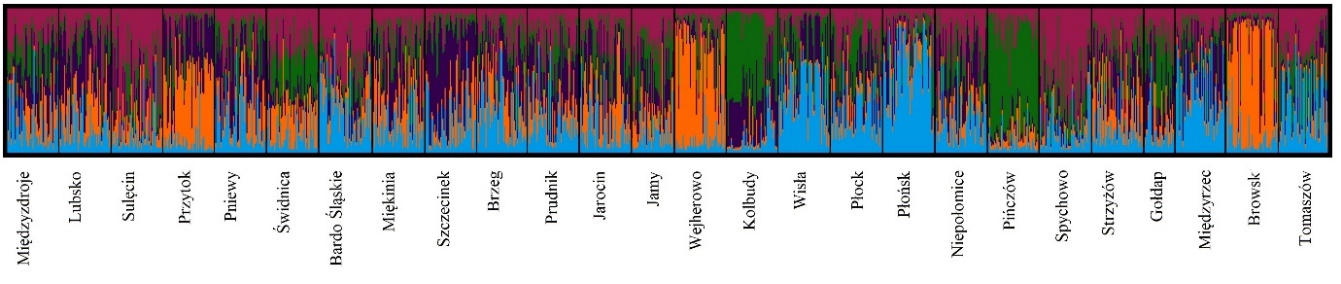 | |
| *K* =10 | 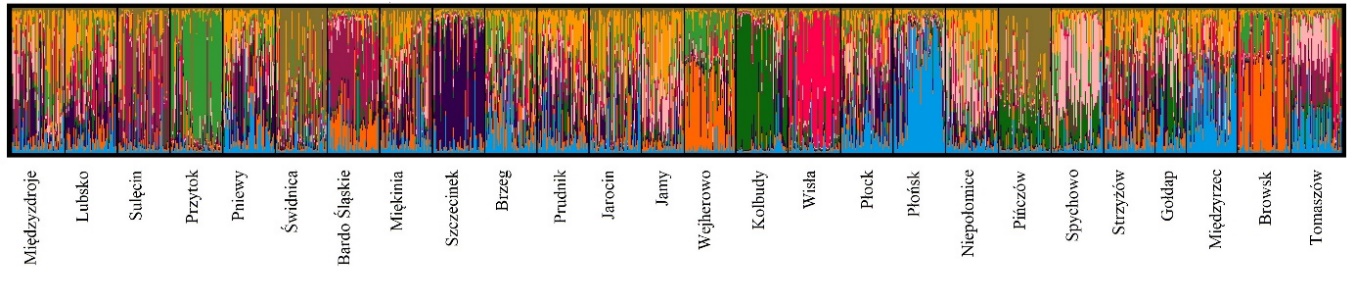 | |

**Figure S3.** Bayesian clustering (STRUCTURE) results for 26 populations of *Fraxinus excelsior* based on nuclear microsatellite loci. (a) The posterior probability of data given each *K* (20 replicates) (mean ± SD), (b) the distribution of delta *K*, (c) assignment plots for *K* = 2, 5 and 10.


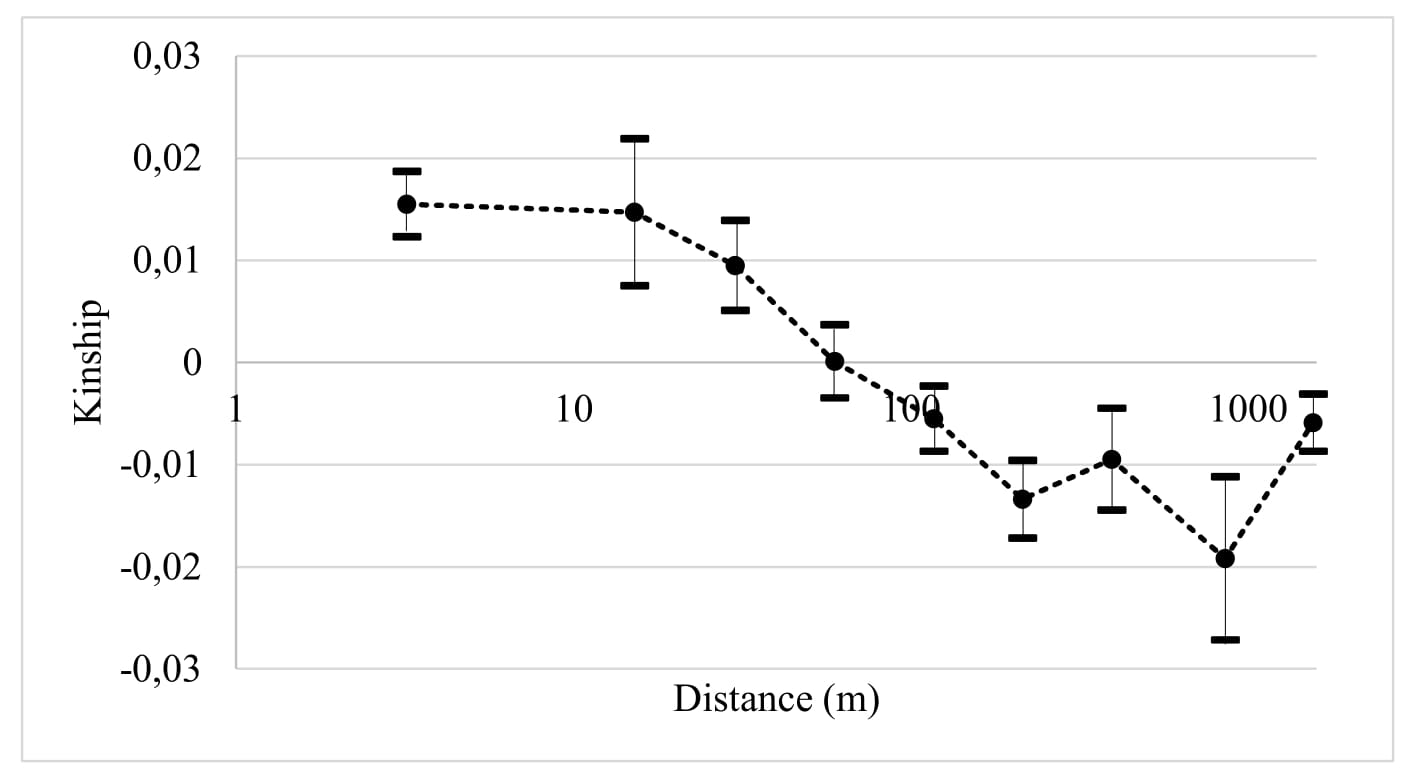


**Figure S4.** Kinship coefficient plotted against the geographical distance between individuals for overall sample. Dots show averages across populations and whiskers indicate the 95% confidence interval estimated based on the standard error (computed using the jackknife across loci).
