# Supplementary material for: Genetic resources of common ash (Fraxinus excelsior L.) in Poland
Source: BMC Plant Biol. 2024 Mar 13;24:186. doi: 10.1186/s12870-024-04886-z (PMC10935948; doi:10.1186/s12870-024-04886-z)
Supplement: Supplementary file 2 — Supplementary Material 2 [file 12870_2024_4886_MOESM2_ESM.docx]

**Table S1.** Characteristics of the haplotypes detected with three polymorphic chloroplast microsatellites in *Fraxinus excelsior*.

| Haplotype | Size of amplified fragment (bp) at chloroplast microsatellites | | | Count | Frequency |
| --- | --- | --- | --- | --- | --- |
|  | ***ccmp5*** | ***ccmp6*** | ***ccmp10*** |  |  |
| *H1* | 101 | 93 | 98 | 738 | 0.582 |
| *H2* | 104 | 91 | 97 | 264 | 0.208 |
| *H3* | 101 | 92 | 98 | 100 | 0.079 |
| *H4* | 102 | 91 | 97 | 81 | 0.064 |
| *H5* | 101 | 93 | 97 | 78 | 0.061 |
| *H6* | 104 | 91 | 98 | 5 | 0.004 |

**Table S2.** The genetic structure of the study populations based on chloroplast microsatellite markers.

| Population ID | Population | *N* | *N_e_* | *GD* | *D* |
| --- | --- | --- | --- | --- | --- |
| 1 | Bardo Śląskie | 1 | 1 | 0 | 0 |
| 2 | Browsk | 3 | 2.47 | 0.607 | 0.585 |
| 3 | Brzeg | 1 | 1 | 0 | 0 |
| 4 | Gołdap | 2 | 1.30 | 0.239 | 0 |
| 5 | Jamy | 2 | 1.16 | 0.139 | 0 |
| 6 | Jarocin | 2 | 1.37 | 0.274 | 0 |
| 7 | Kolbudy | 4 | 1.23 | 0.189 | 0.218 |
| 8 | Lubsko | 1 | 1 | 0 | 0 |
| 9 | Międzyrzec | 2 | 1.99 | 0.509 | 0 |
| 10 | Międzyzdroje | 3 | 2.57 | 0.624 | 0.552 |
| 11 | Miękinia | 1 | 1 | 0 | 0 |
| 12 | Niepołomice | 2 | 1.37 | 0.274 | 0.390 |
| 13 | Pińczów | 2 | 1.92 | 0.49 | 0 |
| 14 | Płock | 2 | 1.22 | 0.184 | 0.300 |
| 15 | Płońsk | 4 | 2.14 | 0.544 | 0.213 |
| 16 | Pniewy | 1 | 1 | 0 | 0 |
| 17 | Prudnik | 1 | 1 | 0 | 0 |
| 18 | Przytok | 3 | 1.23 | 0.189 | 0 |
| 19 | Spychowo | 1 | 1 | 0 | 0 |
| 20 | Strzyżów | 3 | 1.13 | 0.117 | 0.058 |
| 21 | Sulęcin | 2 | 1.85 | 0.47 | 0 |
| 22 | Szczecinek | 2 | 1.04 | 0.04 | 0 |
| 23 | Świdnica | 2 | 1.68 | 0.411 | 0 |
| 24 | Tomaszów | 3 | 1.65 | 0.401 | 0.555 |
| 25 | Wejherowo | 3 | 1.18 | 0.156 | 0.159 |
| 26 | Wisła | 1 | 1 | 0 | 0 |
|  | **Mean** | **2.08** | **1.4** | **0.225** | **0.153** |
|  | SE | 0.18 | 0.09 | 0.042 | 0.038 |

*N* – number of haplotypes, *N_e_* – effective number of haplotypes, *GD* – genetic diversity, *D* – admixture level (based on individual assignment probabilities for K = 2)

**Table S3.** Summary characteristics of ten nuclear microsatellite loci in *Fraxinus excelsior*.

| Locus | *A* | *A_e_* | *A_R_* | *H_o_* | *H_e_* | *F_IS_* | *Null* | *HW* |
| --- | --- | --- | --- | --- | --- | --- | --- | --- |
| *FE4* | 36 | 5.8 | 12.86 | 0.504 | 0.817 | 0.385 | 0.150 | *** |
| *FE8* | 30 | 10.2 | 15.34 | 0.482 | 0.896 | 0.463 | 0.230 | *** |
| *FE11* | 32 | 7.1 | 11.82 | 0.815 | 0.845 | 0.034 | 0.000 | ns |
| *FE12* | 42 | 9.6 | 16.13 | 0.696 | 0.881 | 0.210 | 0.086 | * |
| *FE16* | 10 | 2.3 | 4.64 | 0.239 | 0.544 | 0.554 | 0.191 | * |
| *FE19* | 45 | 10.6 | 15.72 | 0.765 | 0.895 | 0.146 | 0.049 | * |
| *ASH7867* | 11 | 3.0 | 5.72 | 0.617 | 0.653 | 0.057 | 0.003 | ns |
| *ASH53476* | 15 | 1.7 | 5.25 | 0.163 | 0.373 | 0.589 | 0.293 | *** |
| *3.15* | 41 | 8.2 | 15.22 | 0.376 | 0.860 | 0.566 | 0.340 | *** |
| *M2.30* | 57 | 15.4 | 20.73 | 0.760 | 0.925 | 0.177 | 0.071 | ns |
| Mean | **31.9** | **7.4** | **12.34** | **0.542** | **0.769** | **0.318** | **0.141** |  |
| SE | 5.0 | 1.4 | 1.72 | 0.072 | 0.061 | 0.065 | 0.038 |  |

*A* – average number of alleles, *A_e_* – effective number of alleles, *A_R_* – average allelic richness (after rarefaction), *H_o_* – observed heterozygosity, *H_e_* – expected heterozygosity, *Null* – null allele frequency, *F_IS_* – inbreeding coefficient, *HW -* Hardy-Weinberg equilibrium

**Table S4.** Genetic diversity of *Fraxinus excelsior* populations in Poland based on nuclear microsatellite loci.

| Population ID | | Population | | Individuals per population | | Longitude | | Latitude | | *A* | | *A_e_* | | *A_R_* | | *H_e_* | | *H_o_* | | *Null* | | *F_IS_INEST* | | | *F_IS_* | |
| --- | --- | --- | --- | --- | --- | --- | --- | --- | --- | --- | --- | --- | --- | --- | --- | --- | --- | --- | --- | --- | --- | --- | --- | --- | --- | --- |
| 1 | | Bardo Śląskie | | 50 | | E 16° 36' | | N 50° 37' | | 13.2 | | 6.48 | | 11.17 | | 0.546 | | 0.739 | | 0.128 | | 0.044 | | 0.283 | | |
| 2 | | Browsk | | 51 | | E 23° 38' | | N 52° 54' | | 11.6 | | 4.99 | | 10.11 | | 0.513 | | 0.718 | | 0.175 | | 0.028 | | 0.311 | | |
| 3 | | Brzeg | | 50 | | E 17° 22' | | N 50° 48' | | 16.5 | | 8.12 | | 13.6 | | 0.581 | | 0.792 | | 0.133 | | 0.048 | | 0.303 | | |
| 4 | | Gołdap | | 30 | | E 22° 31' | | N 54° 19' | | 13.0 | | 7.79 | | 12.76 | | 0.535 | | 0.772 | | 0.087 | | 0.12 | | 0.339 | | |
| 5 | | Jamy | | 41 | | E 18° 24' | | N 53° 21' | | 13.9 | | 7.95 | | 12.44 | | 0.531 | | 0.756 | | 0.153 | | 0.103 | | 0.285 | | |
| 6 | | Jarocin | | 50 | | E 17° 31' | | N 52° 8' | | 15.4 | | 8.99 | | 13.23 | | 0.52 | | 0.777 | | 0.131 | | 0.144 | | 0.353 | | |
| 7 | | Kolbudy | | 50 | | E 18° 37' | | N 54° 8' | | 13.6 | | 4.95 | | 10.99 | | 0.515 | | 0.754 | | 0.174 | | 0.014 | | 0.322 | | |
| 8 | | Lubsko | | 50 | | E 15° 00' | | N 51° 47' | | 17.3 | | 8.87 | | 14.15 | | 0.564 | | 0.785 | | 0.129 | | 0.061 | | 0.308 | | |
| 9 | | Międzyrzec | | 49 | | E 23° 1' | | N 51° 55' | | 14.1 | | 8.45 | | 12.69 | | 0.563 | | 0.812 | | 0.172 | | 0.105 | | 0.311 | | |
| 10 | | Międzyzdroje | | 50 | | E 14° 32' | | N 53° 58' | | 15.6 | | 8.54 | | 13.30 | | 0.525 | | 0.805 | | 0.136 | | 0.066 | | 0.367 | | |
| 11 | | Miękinia | | 50 | | E 16° 43' | | N 50° 59' | | 17.9 | | 9.57 | | 14.92 | | 0.54 | | 0.794 | | 0.107 | | 0.076 | | 0.344 | | |
| 12 | | Niepołomice | | 50 | | E 20° 22' | | N 52° 7' | | 13.6 | | 7.71 | | 11.86 | | 0.509 | | 0.800 | | 0.179 | | 0.061 | | 0.39 | | |
| 13 | | Pińczów | | 50 | | E 20° 26' | | N 50° 25' | | 11.0 | | 5.07 | | 9.56 | | 0.612 | | 0.729 | | 0.087 | | 0.036 | | 0.195 | | |
| 14 | | Płock | | 50 | | E 20° 9' | | N 52° 28' | | 17.7 | | 9.83 | | 14.72 | | 0.621 | | 0.825 | | 0.135 | | 0.067 | | 0.259 | | |
| 15 | | Płońsk | | 50 | | E 20° 18' | | N 52° 44' | | 14.8 | | 6.48 | | 12.36 | | 0.599 | | 0.782 | | 0.126 | | 0.016 | | 0.249 | | |
| 16 | | Pniewy | | 50 | | E 16° 15' | | N 52° 27' | | 16.9 | | 9.04 | | 14.22 | | 0.573 | | 0.781 | | 0.158 | | 0.059 | | 0.302 | | |
| 17 | | Prudnik | | 49 | | E 17° 28' | | N 50° 30' | | 17.0 | | 9.01 | | 14.09 | | 0.547 | | 0.801 | | 0.153 | | 0.173 | | 0.337 | | |
| 18 | | Przytok | | 50 | | E 15° 33' | | N 52° 1' | | 15.8 | | 9.02 | | 13.46 | | 0.496 | | 0.807 | | 0.114 | | 0.103 | | 0.413 | | |
| 19 | | Spychowo | | 50 | | E 21° 24' | | N 53° 34' | | 10.9 | | 6.61 | | 9.86 | | 0.587 | | 0.768 | | 0.129 | | 0.042 | | 0.266 | | |
| 20 | | Strzyżów | | 50 | | E 21° 51' | | N 49° 58' | | 16.5 | | 8.16 | | 13.59 | | 0.52 | | 0.731 | | 0.120 | | 0.076 | | 0.316 | | |
| 21 | | Sulęcin | | 50 | | E 15° 19' | | N 52° 26' | | 13.7 | | 6.02 | | 11.51 | | 0.482 | | 0.732 | | 0.168 | | 0.073 | | 0.383 | | |
| 22 | | Szczecinek | | 50 | | E 16° 33' | | N 50° 54' | | 13.4 | | 7.35 | | 11.51 | | 0.614 | | 0.831 | | 0.129 | | 0.064 | | 0.264 | | |
| 23 | | Świdnica | | 50 | | E 16° 42' | | N 53° 45' | | 14.2 | | 5.84 | | 11.95 | | 0.526 | | 0.726 | | 0.148 | | 0.057 | | 0.291 | | |
| 24 | | Tomaszów | | 50 | | E 23° 33' | | N 50° 17' | | 14.4 | | 6.24 | | 12.19 | | 0.495 | | 0.75 | | 0.211 | | 0.058 | | 0.365 | | |
| 25 | | Wejherowo | | 49 | | E 18° 28' | | N 54° 42' | | 13.9 | | 5.31 | | 11.35 | | 0.479 | | 0.724 | | 0.130 | | 0.056 | | 0.349 | | |
| 26 | | Wisła | | 50 | | E 18° 50' | | N 49° 36' | | 11.2 | | 5.41 | | 9.61 | | 0.491 | | 0.711 | | 0.141 | | 0.048 | | 0.373 | | |
|  | | **Mean** | |  | |  | |  | | **14.5** | | **7.38** | | **12.35** | | **0.542** | | **0.769** | | **0.141** | | **0.069** | | **0.318** | | |
|  | | SE | |  | |  | |  | | 0.404 | | 0.306 | | 0.306 | | 0.008 | | 0.007 | | 0.006 | | 0.007 | | 0.010 | | |

*A* – average number of alleles, *A_e_* – effective number of alleles, *A_R_* – average allelic richness (after rarefaction), *H_o_* – observed heterozygosity, *H_e_* – expected heterozygosity, *Null* – null allele frequency, *F_IS_ INEST* – inbreeding coefficient (estimated using the Bayesian procedure robust to null alleles), *CIF_ISINEST_* – symmetric Bayesian 95% credible interval, *F_IS_* – Wright’s fixation index.

**Table S5.** Spatial genetic structure parameters for *Fraxinus excelsior* using nuclear markers.

| Population ID | Population | *b_log_*(SE) | *F1*(SE) | *Sp* | *N_b_* |
| --- | --- | --- | --- | --- | --- |
| 1 | Bardo Śląskie | -0.009 (0.003)* | 0.064 (0.023)* | 0.010 | 100 |
| 2 | Brzeg | -0.002 (0.001)* | 0.021 (0.012) ns | 0.002 | 500.52 |
| 3 | Gołdap | -0.019 (0.006)* | -0.003 (0.041) ns | 0.019 | 51.62 |
| 4 | Jamy | -0.017 (0.005)* | 0.068 (0.031)* | 0.018 | 55.4 |
| 5 | Kolbudy | -0.006 (0.002)* | 0.051 (0.019)* | 0.006 | 172.53 |
| 6 | Lubsko | -0.008 (0.003)* | 0.001 (0.018) ns | 0.008 | 129.18 |
| 7 | Międzyrzec | -0.004 (0.004) ns | -0.002 (0.013) ns | 0.004 | 270.04 |
| 8 | Miękinia | -0.001 (0.001) ns | -0.015 (0.009) ns | 0.001 | 920.88 |
| 9 | Niepołomice | -0.001 (0.001) ns | 0.003 (0.008) ns | 0.001 | 1542.52 |
| 10 | Pińczów | 0.000 (0.001) ns | -0.016 (0.016) ns | 0.000 | 3584.36 |
| 11 | Płock | -0.002 (0.001)* | 0.011 (0.010) ns | 0.002 | 414.44 |
| 12 | Płońsk | -0.004 (0.001)* | 0.046 (0.018)* | 0.004 | 227.06 |
| 13 | Pniewy | -0.005 (0.001)* | 0.039 (0.024) ns | 0.005 | 190.17 |
| 14 | Prudnik | -0.001 (0.001) ns | 0.009 (0.007) ns | 0.001 | 1052.59 |
| 15 | Przytok | -0.002 (0.002) ns | 0.007 (0.011) ns | 0.002 | 413.01 |
| 16 | Spychowo | -0.005 (0.002)* | 0.049 (0.031) ns | 0.005 | 211.09 |
| 17 | Strzyżów | -0.003 (0.002)* | 0.021 (0.009)* | 0.003 | 337.43 |
| 18 | Sulęcin | -0.007 (0.002)* | 0.034 (0.012)* | 0.007 | 144.85 |
| 19 | Szczecinek | -0.002 (0.001)* | 0.056 (0.021)* | 0.002 | 540.57 |
| 20 | Świdnica | 0.001 (0.001) ns | -0.015 (0.009) ns | 0.001 | 817.53 |
| 21 | Tomaszów | -0.016 (0.004)* | 0.056 (0.038) ns | 0.017 | 58.36 |
| 22 | Wejherowo | 0.000 (0.000) ns | 0.003 (0.004) ns | 0.000 | 2079.04 |
| 23 | Wisła | -0.002 (0.001) ns | 0.018 (0.007)* | 0.002 | 561.11 |
|  | **Mean** | **-0.005 (0.002)** | **0.022 (0.017)** | **0.005 (0.001)**** | **189.8***** |

*b_log_* - regression slope of kinship, *F(1)* - average kinship coefficient between individuals at the first distance class, *Sp* -  intensity of SGS, *Nb* - neighborhood size, statistical significance:***p < 0.001; **p < 0.01; *p < 0.05, n.s. – not significant

**Table S6.** Effective population size of *Fraxinus excelsior*.

| Population ID | Population | *r²* | *r²\|Drift* | *LDNe* | CI2,5 | CI97,5 |
| --- | --- | --- | --- | --- | --- | --- |
| 1 | Bardo Śląskie | 0.0279 | 0.0058 | 55.2 | 45.0 | 69.8 |
| 2 | Browsk | 0.0404 | 0.0172 | 17.1 | 15.0 | 19.5 |
| 3 | Brzeg | 0.0235 | 0.0019 | 177.9 | 119.1 | 330.7 |
| 4 | Gołdap | 0.0452 | 0.0071 | 41.8 | 31.3 | 60 |
| 5 | Jamy | 0.0313 | 0.0035 | 92.9 | 66.0 | 148.6 |
| 6 | Jarocin | 0.0261 | 0.0042 | 77.7 | 63.2 | 99 |
| 7 | Kolbudy | 0.0278 | 0.0052 | 61.8 | 48.1 | 83.2 |
| 8 | Lubsko | 0.0227 | 0.0009 | 356.1 | 187.8 | 2240.8 |
| 9 | Międzyrzec | 0.0297 | 0.0055 | 57.9 | 47.8 | 72.1 |
| 10 | Międzyzdroje | 0.0334 | 0.0117 | 26.4 | 23.6 | 29.6 |
| 11 | Miękinia | 0.0242 | 0.0014 | 237.2 | 148.7 | 539.6 |
| 12 | Niepołomice | 0.0252 | 0.0030 | 106.4 | 79.1 | 156.7 |
| 13 | Pińczów | 0.0287 | 0.0070 | 45.6 | 37.2 | 57.3 |
| 14 | Płock | 0.0247 | 0.0018 | 182.6 | 121.3 | 346.7 |
| 15 | Płońsk | 0.0262 | 0.0041 | 78.6 | 62.0 | 104.7 |
| 16 | Pniewy | 0.0265 | 0.0027 | 121.6 | 88.7 | 186.2 |
| 17 | Prudnik | 0.0231 | 0.0011 | 308.2 | 171.1 | 1239.4 |
| 18 | Przytok | 0.0255 | 0.0025 | 129.5 | 92.4 | 207.0 |
| 19 | Spychowo | 0.0363 | 0.0141 | 21.4 | 18.7 | 24.7 |
| 20 | Strzyżów | 0.0233 | 0.0002 | +∞ | 319.6 | +∞ |
| 21 | Sulęcin | 0.0316 | 0.0092 | 34.0 | 29.1 | 40.2 |
| 22 | Szczecinek | 0.0269 | 0.0049 | 66.7 | 53.0 | 87.7 |
| 23 | Świdnica | 0.0258 | 0.0037 | 85.7 | 65.9 | 118.9 |
| 24 | Tomaszów | 0.0310 | 0.0069 | 46.5 | 38.4 | 57.8 |
| 25 | Wejherowo | 0.0251 | 0.0023 | 14.1 | 94.4 | 293.7 |
| 26 | Wisła | 0.0311 | 0.0084 | 37.3 | 30.8 | 46.1 |
|  | **Mean** | **0.0278** | **0.0052** | **57.5** | **47.76** | **73.8** |

*r^2^* – linkage disequilibrium, *r²|Drift* - linkage disequilibrium with sample correction, *LDNe* – effective population size, *CI2.5* and *CI97.5* – 95% confidence interval

**Table S7.** Description of sampled populations.

| Population ID | Population | Individuals per population | Longitude | Latitude |
| --- | --- | --- | --- | --- |
| 1 | Bardo Śląskie | 50 | E 16° 36' 17.88" | N 50° 37' 3" |
| 2 | Browsk | 51 | E 23° 38' 4.56" | N 52° 53' 54.24" |
| 3 | Brzeg | 50 | E 17° 21' 48.06" | N 50° 47' 34.7" |
| 4 | Gołdap | 30 | E 22° 31' 27.94" | N 54° 19' 12.78" |
| 5 | Jamy | 41 | E 18° 23' 52.94" | N 53° 21' 20.22" |
| 6 | Jarocin | 50 | E 17° 30' 31.08" | N 52° 7' 32.04" |
| 7 | Kolbudy | 50 | E 18° 37' 18.93" | N 54° 7' 35.39" |
| 8 | Lubsko | 50 | E 14° 59' 38.49" | N 51° 47' 22.04" |
| 9 | Międzyrzec | 49 | E 23° 1' 14.08" | N 51° 54' 56.81" |
| 10 | Międzyzdroje | 50 | E 14° 31' 52.38" | N 53° 57' 40.92" |
| 11 | Miękinia | 50 | E 16° 43' 29.01" | N 50° 58' 32.47" |
| 12 | Niepołomice | 50 | E 20° 21' 36.16" | N 52° 6' 38.59" |
| 13 | Pińczów | 50 | E 20° 26' 22.71" | N 50° 24' 54.94" |
| 14 | Płock | 50 | E 20° 8' 52.87" | N 52° 28' 29.93" |
| 15 | Płońsk | 50 | E 20° 18' 13.41" | N 52° 44' 23.01" |
| 16 | Pniewy | 50 | E 16° 15' 0.89" | N 52° 26' 38.68" |
| 17 | Prudnik | 49 | E 17° 27' 46.47" | N 50° 30' 11.32" |
| 18 | Przytok | 50 | E 15° 32' 30.19" | N 52° 1' 23.12" |
| 19 | Spychowo | 50 | E 21° 23' 59.27" | N 53° 34' 0.21" |
| 20 | Strzyżów | 50 | E 21° 51' 8.26" | N 49° 57' 34.64" |
| 21 | Sulęcin | 50 | E 15° 18' 35.83" | N 52° 25' 47.39" |
| 22 | Świdnica | 50 | E 16° 33' 21.73" | N 50° 54' 21.97" |
| 23 | Szczecinek | 50 | E 16° 42' 21.06" | N 53° 44' 30.57" |
| 24 | Tomaszów | 50 | E 23° 33' 11.59" | N 50° 17' 10.92" |
| 25 | Wejherowo | 49 | E 18° 27' 39.52" | N 54° 41' 32.67" |
| 26 | Wisła | 50 | E 18° 50' 7.26" | N 49° 36' 29.12" |

**Table S8.** Details about the optimized multiplex –PCR reaction mixture.

| Component | Concentration |
| --- | --- |
| Multiplex 1 | |
| Qiagen Multiplex PCR master mix (Qiagen) | 1x |
| BSA | 0,5 mg/ml |
| *Ccmp2* | 50nM |
| *Ccmp5* | 150nM |
| *Ccmp6* | 50nM |
| *Ccmp10* | 50nM |
| *µkk2* | 150nM |
| DNA | 10 ng |
| Multiplex 2 | |
| Qiagen Multiplex PCR master mix (Qiagen) | 1x |
| BSA | 0,5 mg/ml |
| *Femsatl4* | 50nM |
| *Femsatl12* | 75nM |
| *Femsatl16* | 100nM |
| *Femsat19* | 50nM |
| *ASH7867* | 25nM |
| DNA | 10 ng |
| Multiplex 3 | |
| Qiagen Multiplex PCR master mix (Qiagen) | 1x |
| BSA | 0,5 mg/ml |
| *Femsatl8* | 150nM |
| *Femsatl10* | 125nM |
| *M2.30* | 100nM |
| *3.15* | 125nM |
| *ASH53476* | 25nM |
| DNA | 10 ng |
